# An Unconventional Flavivirus and Other RNA Viruses in the Sea Cucumber (Holothuroidea; Echinodermata) Virome

**DOI:** 10.3390/v12091057

**Published:** 2020-09-22

**Authors:** Ian Hewson, Mitchell R. Johnson, Ian R. Tibbetts

**Affiliations:** 1 Department of Microbiology, Cornell University, Ithaca, NY 14853, USA; mrj78@cornell.edu; 2 School of Biological Sciences, University of Queensland, St Lucia, Brisbane, QLD 4072, Australia; i.tibbetts@uq.edu.au

**Keywords:** holothurian, *Apostichopus*, wasting, virus, flavivirus, totivirus

## Abstract

Sea cucumbers (Holothuroidea; Echinodermata) are ecologically significant constituents of benthic marine habitats. We surveilled RNA viruses inhabiting eight species (representing four families) of holothurian collected from four geographically distinct locations by viral metagenomics, including a single specimen of *Apostichopus californicus* affected by a hitherto undocumented wasting disease. The RNA virome comprised genome fragments of both single-stranded positive sense and double stranded RNA viruses, including those assigned to the *Picornavirales*, *Ghabrivirales*, and *Amarillovirales*. We discovered an unconventional flavivirus genome fragment which was most similar to a shark virus. *Ghabivirales*-like genome fragments were most similar to fungal totiviruses in both genome architecture and homology and had likely infected mycobiome constituents. *Picornavirales*, which are commonly retrieved in host-associated viral metagenomes, were similar to invertebrate transcriptome-derived picorna-like viruses. The greatest number of viral genome fragments was recovered from the wasting *A. californicus* library compared to the asymptomatic *A. californicus* library. However, reads from the asymptomatic library recruited to nearly all recovered wasting genome fragments, suggesting that they were present but not well represented in the grossly normal specimen. These results expand the known host range of flaviviruses and suggest that fungi and their viruses may play a role in holothurian ecology.

## 1. Introduction

Next generation DNA sequencing technology applied to viral metagenomics has enabled surveillance of viruses associated with invertebrate tissues. These studies, along with the mining of metazoan transcriptomes, have led to the discovery of novel viral lineages in aquatic invertebrates and broadened the host range of several viral families [1,2,3,4,5,6,7,8,9,10,11,12,13,14,15]. While the majority of viral surveillance and discovery is focused on grossly normal individual specimens of aquatic metazoa, viral metagenomics has been used to examine the presence of potential pathogenic viruses through comparative asymptomatic/disease affected individuals [16]. Despite a growing appreciation of the diversity of aquatic metazoan-associated viruses, and of their potential roles in host disease, they remain largely under-sampled. Study of novel and/or highly divergent viral genomes across a wider range of aquatic invertebrates may provide clues to viral evolution and potential roles in host ecology.

Holothurians (Holothuroidea; Echinodermata) are ecologically important echinoderms [17,18] that can dominate benthic biomass, and contribute significantly to biogeochemical cycling in benthic compartments. On the abyssal plains, for example, holothurian biomass exceeds all other invertebrates [19]. Holothurians may be planktivorous, herbivorous or deposit feeders, and contribute to ecosystem function by regeneration of particulate organic matter into dissolved organic and inorganic constituents [18,20]. Feeding by holothurians on sediments stimulates bacterial production [20] and, potentially, bacterial diversity [21]. Holothurians produce pelagic larvae that as meroplankton contribute to herbivory [22,23,24]. Holothurians are also economically significant, since they are fished and aqua-cultured for human consumption [25]. In China, the world’s largest consumer of holothurians, more than 200 kT worth CNY 176M (US$26M) were produced in 2017 [26]. The economic, evolutionary and ecological significance of holothurians make these groups attractive to study for factors influencing their biology and population dynamics, including potentially pathogenic microorganisms. 

Like all echinoderms, holothurians are deuterostomes and are therefore more closely related to chordates than other invertebrate groups. From a viral perspective, their similarity to chordates, and specifically the similarity of cell surface properties that mediate endocytosis and fusion, suggests that they may be infected by similar viral groups. For example, sialic acids, which are used by a variety of viral families to enter cells [27], became prominent late in evolution, especially in deuterostomes, where they play diverse physiological roles [28]. While echinoderms lack adaptive immunity, genome analyses of the echinoid *Strongylocentrotus purpuratus* revealed homologs of vertebrate immune factors [29,30]. Despite these similarities, few studies have assessed the composition of viruses in echinoderm tissues. DNA viruses, including *Piccovirales* [11,12,14] and *Curlivirales* [31,32] were discovered by viral metagenomic studies using amplified (ϕ29-mediated rolling circle replication) material. RNA viral surveys, which demand a different amplification protocol and handling from that of DNA viruses, have detected *Baphyvirales*, *Picornavirales*, *Articulavirales* and *Tolivirales* [13,33] in asteroids. However, RNA viruses in other echinoderm classes have not been investigated by viral metagenomic approaches.

In this study we surveyed RNA viruses in eight holothurian species collected from four distinct geographic locations (Heron Island, Moreton Bay, Salish Sea and Southeast Alaska) and representing four families of holothurians. We found that viruses associated with these tissues were dominated by *Picornavirales* (*Marnaviridae*) and *Ghabrivirales* (*Totiviridae*), but report on the presence of a deeply branched flavivirus in two holothurian species in the northeast Pacific Ocean. We also compared the composition of viruses associated with a grossly normal *Apostichopus californicus* and a specimen that was affected by sea cucumber wasting (a condition that is not extensively documented in the peer-reviewed literature) but found little difference in viral genome representation.

## 2. Materials and Methods 

### 2.1. Specimen Collection

Holothurian specimens were collected at four geographic locations between 2015 and 2017 (Table 1). Specimens were collected by hand in the intertidal zone or by SCUBA Diver (with the exception of *Cucumaria miniata*, which was obtained by rock dredge). Specimens of *Apostichopus californicus* (renamed from *Parastichopus californicus* [34]) were collected by commercial fishers and retrieved by the Alaska Department of Fish and Game. The presence of a wasting-like condition was determined by gross disease signs on collection (see Section 3.4). Specimens were sub-sampled either immediately after collection, or by whole specimens being frozen on dry ice or liquid N_2_ before transport to the laboratory at Cornell University, where they were thawed and dissected prior to metavirome preparation.

### 2.2. Metavirome Preparation

Metaviromes were prepared from each specimen by first retrieving an 8 mm biopsy punch from body wall tissues, which was extruded into a sterile microcentrifuge tube. From here, viral metagenomes were prepared according to established protocols as described in [35] with modifications [36] and omitting the chloroform treatment step. Briefly, tissue samples were disrupted by bead beating (Zymo Bead Beaters) in 2 mL virus-free (i.e., 0.02 µm filtered) PBS, then centrifuged at 5000× *g* for 5 min to remove large particulate material. The resulting supernatant was then removed, filtered through 0.2 µm PES filters (to remove host cells and cell debris), and then treated with RNAseOne (50 U), DNAse I (5 U), and Benzonase nuclease (250 U) for 2 h at 37 °C. Nuclease activity was arrested by amendment with 50 µM EDTA. RNA was extracted from purified virus-sized material using the Zymo Viral RNA Kit. Extracted RNA was then amplified using the TransPlex WTA2 kit (Sigma Aldrich) applied to a 5 µL extract. Because we did not standardize template amounts, and because of uncertainties in bias introduced by TransPlex amplification, data presented in this manuscript are not quantitative and based solely on presence and absence of genome fragments [16]. Amplified DNA (the end product of the TransPlex protocol) was submitted to the Cornell University Biotechnology Resource Center, where it was prepared with the Nextera XT library preparation protocol and sequenced on an Illumina MiSeq 2 × 250 bp platform. Sequence libraries are available at National Center for Biotechnology Information (NCBI) under accession PRJNA417963.

### 2.3. Viral Metagenome Library Analyses

Sequence libraries were first trimmed for ambiguous bases, adapters, TransPlex primers, and poor quality (N < 2) sequences using the Trim Sequences function in the CLC Genomics Workbench 4.0 (Qiagen, Germany). Sequence libraries were then assembled using a minimum overlap of 0.2 and similarity of 0.9 resulting in contig spectra for each library. The resulting contig spectra was aligned against several boutique databases of RNA viruses: (1) all RNA viral library using tBLASTx [37] (genome sequences retrieved in September 2018 from NCBI using search term “RNA Virus”); (2) all *Mononegavirus* proteins (search term “mononegaviruses”) by BLASTx; *Picornavirus* ribosome-dependent RNA polymerase (RdRp) proteins (search term “RdRp AND picornavirus”) by BLASTx; invertebrate RNA viral proteins (search term “invertebrate AND RNA viruses”) by BLASTx; *Flavivirus* proteins (search term “flavivirus”) by BLASTx; *Coronavirus* proteins (search term “coronavirus”) by BLASTx; and nodavirus proteins (search term “nodavirus”) by BLASTx. Sequences were further vetted by comparing matches to viral proteins against the non-redundant database at NCBI by BLASTn, and matches (E < 10^−30^) to bacterial or eukaryotic genes were removed from further consideration. Finally, contigs were compared to the RefSeq and non-redundant protein database by BLASTx to retrieve closest matches from cultivated and uncultivated viruses. Viral contiguous sequences are available at NCBI under accessions MT949664–MT949685.

### 2.4. Viral Genome Architecture and Phylogeny

The open reading frames on vetted viral contigs were extracted with GetORF [38] and compared against the established genome architecture of viral families. If significant matches against proteins were detected, but open reading frame (ORF lengths) were shorter than expected based on reported genome architectures, the sequence was examined for internal frameshifts, which are common in viral ORFs. ORFs were then annotated by comparison against the RefSeq Viral Proteins Database (NCBI). ORFs and partial ORFs adjacent to annotated viral proteins were further compared against the non-redundant database at NCBI by tBLASTx [37] and, if no matches were found, then by the Phyre protein server [39]. Internal secondary RNA structures corresponding with published viral genomes were checked using the mFold server [40]. Additional features were identified by the conserved domain database (CDD) at NCBI [41]. α-helix and β-strand regions of ORFs were determined by PSIPRED [42]. Viral contig sequences and closest matches in NCBI were collected and aligned using the native alignment algorithm in CLC Sequence Viewer 8.0 (gap open cost 10.0; gap extension cost 1; end gap cost as any other), and trimmed to contiguous sequences within protein encoding regions. Phylogenetic representations were performed using both the CLC Sequence Viewer 8.0 and MEGA 10.1.8 [43].

### 2.5. Eukaryotic 18S and 28S rRNA Analyses

18S and 28S rRNAs were identified in viral metagenomes by comparison of contig spectra against the Silva database [44] by BLASTn (E < 10^−25^). Contigs matching this criterion were further annotated based on nearest match in NCBI by BLASTn, and classified based on taxonomic descriptions provided by the NCBI Taxonomy Database. 

## 3. Results and Discussion 

Twenty-two contiguous sequences met our criteria as matching viral proteins (from here referred to as “viral contigs”), which were primarily within the *Picornavirales* (*Marnaviridae* and *Dicistroviridae*, referred to from here as “picornavirus-like”), *Ghabrivirales* (*Totiviridae*, referred to from here as “totivirus-like”), and a single contig matching the *Amarillovirales* (*Flaviviridae*, referred to from here as “flavivirus-like”). The *Apostichopus californicus* wasting library yielded the most viral contigs (Table 1; *n* = 11), followed by *Cucumaria miniata* and *Stichopus horrens* (*n* = 3), and single viral contigs in the *Holothuria atra*, *Holothuria scabra* and *Holothuria difficilis* libraries. No viral contigs were retrieved from the *Holothuria pardalis* or *Synaptula recta* libraries. Read recruitment of all libraries against the 22 viral contigs revealed that some were specific to one library, but others were more cosmopolitan. Aside from a single picornavirus-like contig (708), all other picornavirus-like contigs were present in only the *C. miniata* and *A. californicus* libraries. Conversely, of the five totivirus-like contigs, only two were specific to a single library, and two (contigs 5835 and 4411) recruited from both Australian and North American holothurians. The flavivirus-like contig (91) recruited from only the North American holothurian libraries, in both *C. miniata* and *A. californicus*. Comparing the composition of wasting and grossly normal *A. californicus*, all contigs except one (picornavirus-like contig 1223) that were assembled in the wasting affected specimen also recruited reads from the grossly normal specimen library.

These results indicate that most RNA viruses discovered in this survey were restricted to a single or perhaps sympatric holothurian species, with few that spanned species or genera. Viral host tropism is circumscribed by inherent cell properties, including those affecting entry, replication and release/shedding of virions. Similar species may exhibit greater shared tropism for viruses, which may result in spillover events of pathogens [45]. While variation in factors affecting tropism may result in restricted host range [46], viruses may also infect across highly unrelated hosts, e.g., arboviruses that infect both insects and vertebrates. Amongst the 22 viral contigs retrieved in this survey, genome architecture and features suggest variable hosts, which may include non-holothurian microorganisms and metazoa. Hence, it is not possible to speculate on their host range. However, our data demonstrate that some viral genotypes may have geographically widespread distribution, since contigs recruited reads from libraries generated from Australian and North American specimens (Table 2).

### 3.1. Flavivirus-Like Genome Fragment

**Figure 1:**
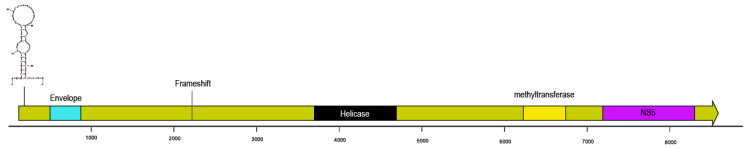
Contig map of *Apostichopus californicus* flavivirus-like contig 91. The open reading frame matched a flavivirus polyprotein by BLASTx [37]. Methyltransferase, NS5 and helicase domains were identified by comparison against the conserved domain database (CDD) at NCBI [41]. The location of the envelope region was determined by protein folding comparison in Phyre [39]. The hairpin like structure preceding the Envelope region was determined by folding all sites between start (AUG) codons by mFold [40].

A single flavivirus-like contig (*A. californicus* contig 91) was retrieved based on homology with flaviviruses deposited at NCBI. This 8883 nt contig bore a single open reading frame (ORF; 8318 nt; flavivirus polyprotein by CDD search), with 391 nt untranslated region (UTR) at the 5′-end. Within the polyprotein ORF, a region corresponding to the NS5 of flavivirus RNA-dependent RNA polymerase (RdRp) was observed at the 3′ end. Preceding this is a conserved helicase domain (comprising DEAD N- and C-terminus motifs flanking a Walker motif) but at the 5′ end there was no similarity based on homology with any known flavivirus. However, analysis of this region by predicted peptide folding revealed a high confidence match to flavivirus envelope protein (nt positions ~400–700; confidence = 86.7; crystal structure of the envelope glycoprotein ectodomain from dengue2 virus serotype 4), suggesting it was distant from known representative flaviviruses. Within this region, there was a slippery sequence frameshift at position 2061 (5′-CUCCCUUUUUUAUC-3′) which is also observed in insect-specific flaviviruses [47]. A start codon (AUG)-flanked hairpin structure, which is characteristic of flaviviruses is at nt positions 232–274 [48,49] (Figure 1). There was no similarity to known capsid or membrane proteins at the 3′ end based on CDD search or predicted peptide folding. The first 1000 amino acids contained mostly β helices, whereas the region closest to the RdRp bore more α-helices, suggesting it may bear the structural region [50]. The genome arrangement of the *A. californicus* flavivirus-like genome fragment is similar to other insect-specific flaviviruses and distinct from flavivirus arboviruses. Reads recruited from the *C. miniata* library to this flavivirus-like genome, but not from other holothurian species (Table 2). 

**Figure 2:**
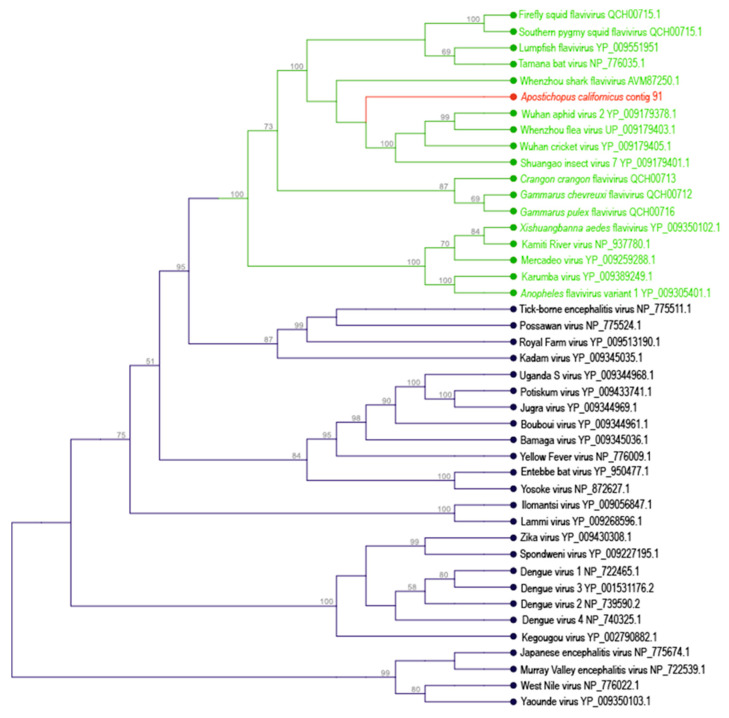
Phylogenetic representation of *Apostichopus californicus* flavivirus-like contig 91. The tree was constructed by performing an alignment of overlapping regions with best BLASTx matches at NCBI using the CLC Sequence Viewer 8.0 native alignment algorithm. The tree is based on a ~420 amino acid alignment by neighbor joining and based on Jukes-Cantor distance. Values above nodes indicate bootstrap statistics (>50%) based on 1000 iterations. The green branches indicate the emerging aquatic and invertebrate-associated flavivirus clade [52]. An additional phylogenetic representation based on maximum likelihood is provided in the Supplementary Materials.

Alignment of the *A. californicus* flavivirus-like genome fragment against closest matches at NCBI revealed that it was firmly embedded within a clade of insect-specific flaviviruses [51], including flaviviruses recovered from cephalopods [52] and from invertebrate and vertebrate transcriptomes [53] (Figure 2). Enveloped RNA viruses are notable in marine ecosystems because they are not common constituents of virioplankton and generally experience high decay rates in seawater [54]. Flaviviruses (+ssRNA) represent important pathogens of mammals, including Dengue Fever, Yellow Fever, Hepatitis C Virus and Zika Virus. While most flaviviruses recovered to date infect mammals and represent arboviruses (arthropod-borne viruses) that cause pathology in mammals but do not cause disease in arthropod vectors, there is an expanding clade of flaviviruses that are found only in arthropods and never in vertebrates [55]. These viruses have been termed “insect-specific flaviviruses (iFVs)”. Despite this moniker, iFVs have also recently been observed in teleosts [56,57], crustaceans [52] and an elasmobranch [53]. Our discovery of a flavivirus genome fragment associated with a holothurian host expands the host range attributed to this group and suggests that these may be more widespread in invertebrate groups than currently understood. Parry and Asgari [52] inferred circulation of flaviviruses between vertebrates and invertebrates based on nucleotide frequencies and active interference RNA response to a shark flavivirus in crabs. The observation of vertebrate-invertebrate flavivirus associations in marine habitats and in terrestrial habitats may suggest they have arisen twice. Because flavivirus host range restriction occurs at multiple levels [48], vertebrate-invertebrate associations have evolved through complex interactions in order to overcome barriers to infection and replication. Our observation of a holothurian flavivirus that has greatest similarity to a shark flavivirus [53] suggests that either these are unique to deuterostomes, or that these may represent similar invertebrate-vertebrate viral associations. 

### 3.2. Picornavirales-Like Genome Fragments

**Figure 3:**
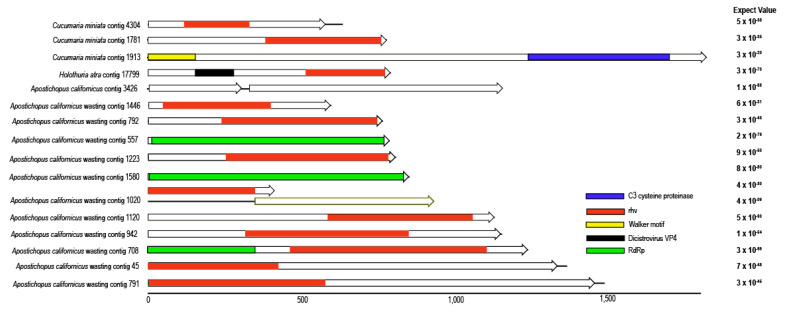
Contig maps for *Picornavirales*-like genome fragments recovered from holothurians by viral metagenomics. The expect values of best matches by BLASTx against the non-redundant database at NCBI are indicated to the right of each contig. Solid lines indicate the total contig length, and arrows indicate open reading frames. Colored bars indicate shared homology between contigs based on reciprocal tBLASTx.

**Figure 4:**
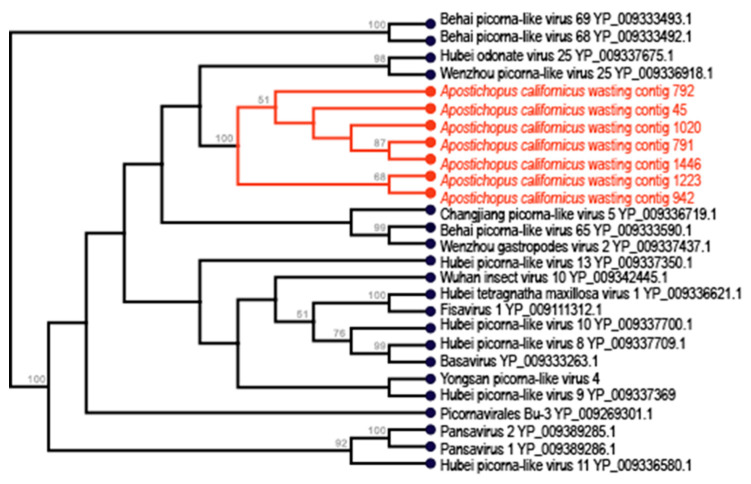
Phylogenetic representation of holothurian-associated *Picornavirales*-like genome fragments. The tree was constructed by performing an alignment of an overlapping region (~100 amino acid) of the rhv domain with best BLASTx matches in the non-redundant database at NCBI. The tree was constructed by neighbor joining and based on Jukes-Cantor distance. Values above nodes indicate bootstrap statistics (>50%) based on 1000 iterations. An additional phylogenetic representation based on maximum likelihood is provided in the Supplementary Materials.

Sixteen contigs were most similar to *Picornavirales* (which comprises picornaviruses, dicistroviruses and iflaviruses) based upon sequence homology to representatives at NCBI (Figure 3). Of these, 11 were retrieved from the wasting-affected *A. californicus* library, while only one contig was retrieved from the grossly normal *A. californicus* library. In addition to *A. californicus* picornavirus-like genome fragments, 3 fragments were also retrieved from *C. miniata*. All contigs bore similarity to RdRp domains of picornavirus polyproteins. *Apostichopus californicus* contig 1020 bore 2 overlapping ORFs, which is uncharacteristic of picornaviruses and may indicate the presence of a frameshift in the overlapping region. Of the 17 picornavirus-like contigs, 11 bore similarity to rhinovirus capsid (rhv) regions, six bore similarity to reverse transcriptase (RT)-like RdRp domains (one contig, from wasting-affected *A. californicus*, contig 708, contained both rhv and RdRp domains), and one contig bore similarity to a Walker motif (P-loop-NTPase) and to the 3C cysteine protease (picornarin). Of particular note, *Holothuria atra* contig 17,799 bore a capsid protein motif most similar to VP4 of Cricket paralysis virus, suggesting that it may belong to the *Dicistroviridae*. Picornaviruses have conserved genome arrangement in the order rhv-helicase-protease-polymerase [58,59,60]. Interestingly, the one contig bearing both rhv and RdRp motifs was in the arrangement from 5′- to 3′- that was opposite to conserved picornaviruses and iflaviruses and more similar to dicistroviruses [60]. Phylogenetic analyses of detected *Picornavirales* based around the rhv domain demonstrate that they are similar to picorna-like viruses retrieved from transcriptomes of insects, suggesting an invertebrate host (Figure 4). 

Picornavirus-like genomes are routinely recovered in invertebrate host-associated and environmental RNA viral metaviromes [12,33,61,62,63,64,65,66,67,68]. There have been no previous reports of picornaviruses associated with any disease in aquatic invertebrates, and their pattern of association between sea star wasting (SSW)-affected and grossly normal asteroid specimens does not suggest their involvement in disease process [33]. *Picornavirales* infect a wide range of hosts including metazoa and unicellular eukaryotes [69]. Hence, it is possible that some detected picornavirus-like viruses may infect microscopic eukaryotic constituents of the holothurian microbiome. Our observation of a dicistrovirus-like genome fragment in *H. atra* may suggest that this infects the holothurian. The known host range of dicistroviruses includes only arthropods and other invertebrates [62]. Similarly, the phylogenetic similarity of rhv domain-bearing contigs in this survey suggest an invertebrate host rather than unicellular eukaryotic hosts.

### 3.3. Totivirus-Like Genome Fragments

**Figure 5:**
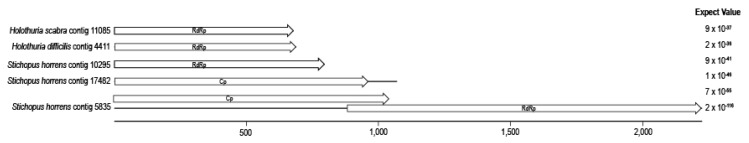
Contig maps for totivirus-like genome fragments recovered from holothurians. The expected values of best matches by BLASTx [37] to the non-redundant database at NCBI are indicated to the right of the contigs. Solid lines indicate the total contig length, and arrows indicate open reading frames. RdRp = RNA-dependent RNA polymerase, Cp = capsid protein.

**Figure 6:**
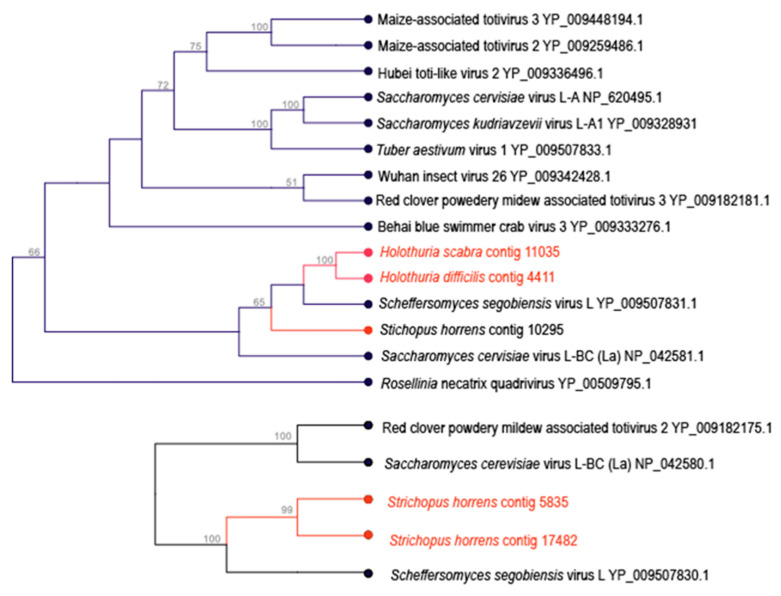
Phylogenetic representations of holothurian-associated totivirus-like genome fragments. The trees were constructed by performing an alignment of an overlapping region of the RdRp (**top**) and Cp (**bottom**) domains with best BLASTx matches at NCBI. The trees are based on ~156 amino acid (for RdRp) and 87 amino acid (for Cp) alignments by neighbor joining and based on Jukes-Cantor distance. Values above nodes indicate bootstrap statistics (>50%) based on 1000 iterations. An additional phylogenetic representation based on maximum likelihood is provided in the Supplementary Materials.

Five contiguous sequences matching totivirus genome fragments were recovered from three species of holothurian, with most coming from *Stichopus horrens* (Figure 5). These represented RdRps (*n* = 4) and capsid (Cp) proteins (*n* = 2), with one contig (*S. horrens* contig 5835) bearing an overlapping Cp-RdRp region, with a possible frameshift at position 882, characteristic of totivirus ORFs [70]. Predicted secondary structure (PSIPRED V4.0 [71]) of the *S. horrens* contig 5835 major capsid protein region revealed a 5′ α- and β-strand rich region, followed by a β-helix rich region at the 3′ end, which is most similar to fungal totiviruses and less similar to metazoan totiviruses, which have an α-helix rich 3′ region [72]. The Cp on *S. horrens* contig 17482, on the other hand, bears an α- and β-rich region at the 5′ end, followed by a β-rich region and terminating in an α-rich region, which suggests it may be more closely related to arthropod totiviruses [72]. Phylogenetically, the Cp regions on both *S. horrens* 5835 and 17,482 match more closely fungal totivirus Cp regions, as do all RdRp fragments recovered in this study (Figure 6). Hence, these are likely fungal viruses.

Fungi do not comprise a large portion of free-living aquatic protistan communities, but they are frequently found in association with invertebrates [73] and are implicated in at least one invertebrate disease [74]. Fungi are easily cultivated from echinoderms, and especially holothurian tissues [75,76,77], and extracts of holothurian body wall bear antifungal substances [78,79]. To date there have been no cultivation-independent assessments of holothurian microbiome composition. Because viral metagenomes enrich particles <0.2 µm, which includes ribosome-sized particles, rRNAs can comprise a large fraction of metaviromic libraries, even after RNAse treatment to remove co-extracted host RNAs. We probed the phylogeny of 18S and 28S rRNAs recovered in RNA viromes and discovered that most bore signatures of basidiomycete and ascomycete rRNAs (Table 3). Hence, it is not surprising to retrieve fungal viruses in our survey. Nerva et al. [80] observed the diversity of viruses in a fungal collection prepared from *Holothuria polii*. They discovered that more than half were double-stranded RNA (dsRNA) viruses belonging to the *Partitiviridae* and *Chrysoviridae*, the latter of which belongs to the *Ghabrivirales*. Our observation of putatively fungal totiviruses in echinoderm viromes raises interesting questions about the role of fungi, and interactions between these and their viruses in host health and ecology. 

### 3.4. Viral Diseases in Holothurians

A condition affecting *A. californicus* has been observed since at least 2014 in southeast Alaska and the Salish Sea. The ongoing sea star wasting (SSW) epidemic brought attention to the condition in sea cucumbers, which has not yet been reported in the peer-reviewed scientific literature. Because this species primarily inhabits subtidal habitats and is generally not as abundant as asteroids, the extent of sea cucumber wasting (SCW) and impacts on population decline is driven by wildlife manager and fisher observations (cf. extensive citizen science observations of SSW). Wasting was first anecdotally reported in Friday Harbor, WA, in February 2014, subsequently near Admiralty Island (AK) in August 2014, and Santa Catalina Island in September 2014. In April 2015 and again in November 2016, more extensive SCW was observed in the holothurian fishery in southeast Alaska (Tenakee Springs, Chicago Island) and in the central Salish Sea [81]. In February 2017, SCW was observed at an aquaculture facility in the southern Salish Sea (Manchester, WA) and again in the central Salish Sea and has since been reported more widely in the region (including in the Secheldt and Howe Sound, BC). Anecdotally reported disease signs based on gross observations include non-focal lesions and fissures across the body wall, some sloughing of epidermal tissues, and rapid liquefaction upon collection [81]. Anecdotally, the number of individuals recovered at fishing sites decreased from 2014 to the present day, suggesting that SCW may have affected overall holothurian population density. Wasting was mostly reported between September and January in 2014–2017, which corresponds with seasonal evisceration which is a response to more limited food availability and organ atrophy [82]. During seasonal self-evisceration, viscera are recycled by internal processes [83]. However, self-evisceration does not typically affect body wall tissues, so it is unclear whether SCW is an extension of normal self-evisceration processes. 

We compared a single wasting *A. californicus* specimen virome to a single grossly normal *A. californicus* virome collected at the same site (Table 1). We expected to see differential representation of wasting-associated viruses in *A. californicus* which would provide targets for further pathology investigations. However, nearly all viral genome fragments discovered in the wasting-affected individual recruited reads from the asymptomatic specimen library, which is similar to metagenomic analyses performed on sea star wasting-affected individuals [12]. Because of uncertain amplification biases and variable amplification template amounts, it is not possible to quantitatively compare representation between tissue states [16]. Moreover, no challenge experiments with virus-sized material or isolated viruses were performed in this study. Hence, it would be highly speculative with the available information to attribute sea cucumber wasting to any virus.

Previous work has highlighted several virus-like particles associated with tissues of grossly diseased specimens of the aqua-cultured *Apostichopus japonicus*. A skin ulceration and evisceration-associated disease caused mass mortality in the Yellow Sea in 2004–2005. Occluded viral particles were observed by electron microscopy in muscles lining the water vascular system, alimentary canal, connective tissue and respiratory tree, although the virus was not sequenced [84]. In addition, exposure to virus-sized material yielded disease signs in naïve specimens. Separately, [85] reported microscopy-based assessments of acute peristome edema disease in the Yellow Sea in 2007, and described particles with consistent morphology to coronaviruses. Similarly, a spherical virus with a bilayer capsule was described in holothurians experiencing skin ulceration and peristome tumescence disease, and again this appeared to be transmissible [86]. Similar virus particles were also reported in *A. japonicus* larvae experiencing stomach atrophy, which were identical to virus particles observed in parent gonads [84]. 

The eight species of holothurian sampled in this study were broadly similar in ecology. Specimens of *A. californicus*, *S. horrens*, *H. atra*, *H. scabra*, *H. difficilis*, *H. pardalis*, and *S. recta* are deposit feeders that consume detritus and sediment-bound organisms. *A. californicus* inhabits subtidal waters in the northeastern Pacific, whereas other species inhabit primarily subtropical and tropical waters of the Indo-Pacific. *C. miniata* is a suspension feeder that occurs in rocky intertidal zones in the northeastern Pacific. In the context of virology, there were no expected differences due to behavior (which may affect contact rates between individuals or with fomites), except for perhaps between *C. miniata* and other species. However, our observation of read recruits between viral genome fragments detected in *C. miniata* and *A. californicus* suggests that geographic location may ultimately influence viral types present in species more strongly than feeding behavior.

## 4. Conclusions

To the best of our knowledge, this is the first viral metagenomic survey of holothurians, and our work expands on knowledge of viruses inhabiting echinoderms. Our work revealed the presence of a flavivirus-like genome fragment that falls within an expanding group of aquatic invertebrate flaviviruses, along with novel totivirus and picornavirus-like fragments. While we cannot associate pathology with any of these detected viral genotypes and our comparison is limited to a single individual of sea cucumber wasting, we did not observe a viral genotype that was unique to SCW. It is recommended that future studies focus on the entry, replication and shedding of novel flaviviruses and the role of fungi and their associated viruses in holothurian ecology.

## Figures and Tables

**Table 1:** Specimens collected for viral metagenomics survey and library characteristics.

Species	Family	Sample Location	Latitude	Longitude	Collection Date	Sequence Library Size (reads)	# Contigs	N50 Contigs	Contigs Matching Viral Genomes E < 10^−20^	Reads Mapped to Viral Contigs
*Cucumaria miniata*	Cucumariidae	Salish Sea	48.2333 N	122.7917 W	7 January 2016	1,686,849	5394	811	3	247,494
Apostichopus californicus *	Stichopodidae	Ketchikan	55.4351 N	131.9481 W	26 October 2016	897,618	3431	804	11	195,230
*Apostichopus californicus*	Stichopodidae	Ketchikan	55.4351 N	131.9481 W	26 October 2016	1,019,071	3900	880	2	179,175
*Holothuria scabra*	Holothuridae	Amity Banks	27.4115 S	153.4344 E	10 December 2015	822,244	21,777	838	1	368
*Synaptula recta*	Synaptidae	Dunwich	27.4952 S	153.3985 E	10 December 2015	983,151	19,760	847	0	299
*Holothuria difficilis*	Holothuridae	Amity Banks	27.4115 S	153.4344 E	10 December 2015	794,579	5803	846	1	301
*Stichopus horrens*	Stichopodidae	Amity Banks	27.4115 S	153.4346 E	10 December 2015	1,577,532	21,194	817	2	423
*Holothuria atra*	Holothuridae	Heron Island	23.4441 S	151.9113 E	19 December 2015	875,840	18,866	793	1	345
*Holothuria pardalis*	Holothuridae	Dunwich	27.4952 S	153.3985 E	10 December 2015	1,467,795	7424	896	0	306

The * indicates that the specimen was affected by sea cucumber wasting (SCW).

**Table 2:** Read recruitment to metagenome-assembled viral genome fragments across all libraries. + indicates that reads recruited from the metavirome library.

Species of Metagenome	Contig #	Viral Order	Contig Length (nt)	Holothurian Species Recruited								
				Sh	Ha	Hs	Hp	Hd	Sr	Cm	Ac	Ac *
*Stichopus horrens*	17,482	Ghabrivirales	1070	+								
*Stichopus horrens*	5835	Ghabrivirales	2224	+		+		+	+	+	+	+
*Stichopus horrens*	10,295	Ghabrivirales	796	+	+		+			+	+	
*Holothuria atra*	17,799	Picornavirales	793		+							
*Holothuria scabra*	11,085	Ghabrivirales	679			+						
*Holothuria difficilis*	4411	Ghabrivirales	688	+		+		+			+	
*Cucumaria miniata*	4304	Picornavirales	636							+		
*Cucumaria miniata*	1913	Picornavirales	1826							+		
*Cucumaria miniata*	1781	Picornavirales	782							+		
*Apostichopus californicus*	91	Amarillovirales	8883							+	+	+
*Apostichopus californicus*	3426	Picornavirales	1158								+	+
*Apostichopus californicus* *	942	Picornavirales	1156							+	+	+
Apostichopus californicus *	792	Picornavirales	767								+	+
*Apostichopus californicus* *	791	Picornavirales	1491								+	+
*Apostichopus californicus* *	708	Picornavirales	1241	+	+	+	+	+	+	+	+	+
*Apostichopus californicus* *	557	Picornavirales	789								+	+
*Apostichopus californicus* *	45	Picornavirales	1368								+	+
*Apostichopus californicus* *	1580	Picornavirales	854								+	+
*Apostichopus californicus* *	1446	Picornavirales	597								+	+
*Apostichopus californicus* *	1223	Picornavirales	810									+
*Apostichopus californicus* *	1120	Picornavirales	1132								+	+
*Apostichopus californicus* *	1020	Picornavirales	934								+	+

* indicates wasting-affected individual. Sh = *Stichopus horrens*; Ha = *Holothuria atra*; Hs = *Holothuria scabra*; Hp = *Holothuria pardalis*; Hd = *Holothuria difficilis*; Sr = *Synaptula recta*; Cm = *Cucumaria miniata*; Ac = *Apostichopus californicus*.

**Table 3:** Taxonomic assignment of contigs matching eukaryotic 18S/28S rRNAs. The number of contigs matching each group is indicated, but should be treated with caution because of un-defined biases in template amplification.

Group	Taxonomic Assignment	Holothurian Species								
		*Holothuria pardalis*	*Holothuria difficilis*	*Holothuria scabra*	*Holothuria atra*	*Synaptula recta*	*Stichopus horrens*	*Cucumaria miniata*	*Apostichopus californicus*	*Apostichopus californicus* *
Unicellular Eukaryotes	Alveolata			1			2		6	8
	Apusozoa								2	
	Amoebozoa						2			
	Cryptomonada								1	9
	Heterokonta								1	
	Metamonada								1	
	Viridiplantae	2		2	2	63	11	18	6	1
Fungi	Ascomycota	17	14	6	7	8	17	14	2	21
	Basidiomycota	11	8	6	9	8	13	14	12	16
	Fungi intercae sedis						1	1	2	2
Metazoa	Annelida					1				
	Arthropoda	4	2	4	1	3	4	3	5	5
	Asteroidea						2			
	Chordata	3	1	2	2		4	14	3	3
	Cnidaria	1			1			1		2
	Echinoidea					1				
	Holothuria		1	1		1		3	1	1
	Mollusca		1	2	1					1
	Nematoda							3		
	Phoronida			1						
	Platyhelminthes							1		

The * indicates that the specimen was affected by sea cucumber wasting (SCW).

## Supplementary Materials

The following are available online at https://www.mdpi.com/1999-4915/12/9/1057/s1.
