# Supplementary material for: An Unconventional Flavivirus and Other RNA Viruses in the Sea Cucumber (Holothuroidea; Echinodermata) Virome
Source: Viruses. 2020 Sep 22;12(9):1057. doi: 10.3390/v12091057 (PMC7551563; doi:10.3390/v12091057)
Supplement: Supplementary file 1 [file viruses-12-01057-s001.pdf]

Supplemental Material

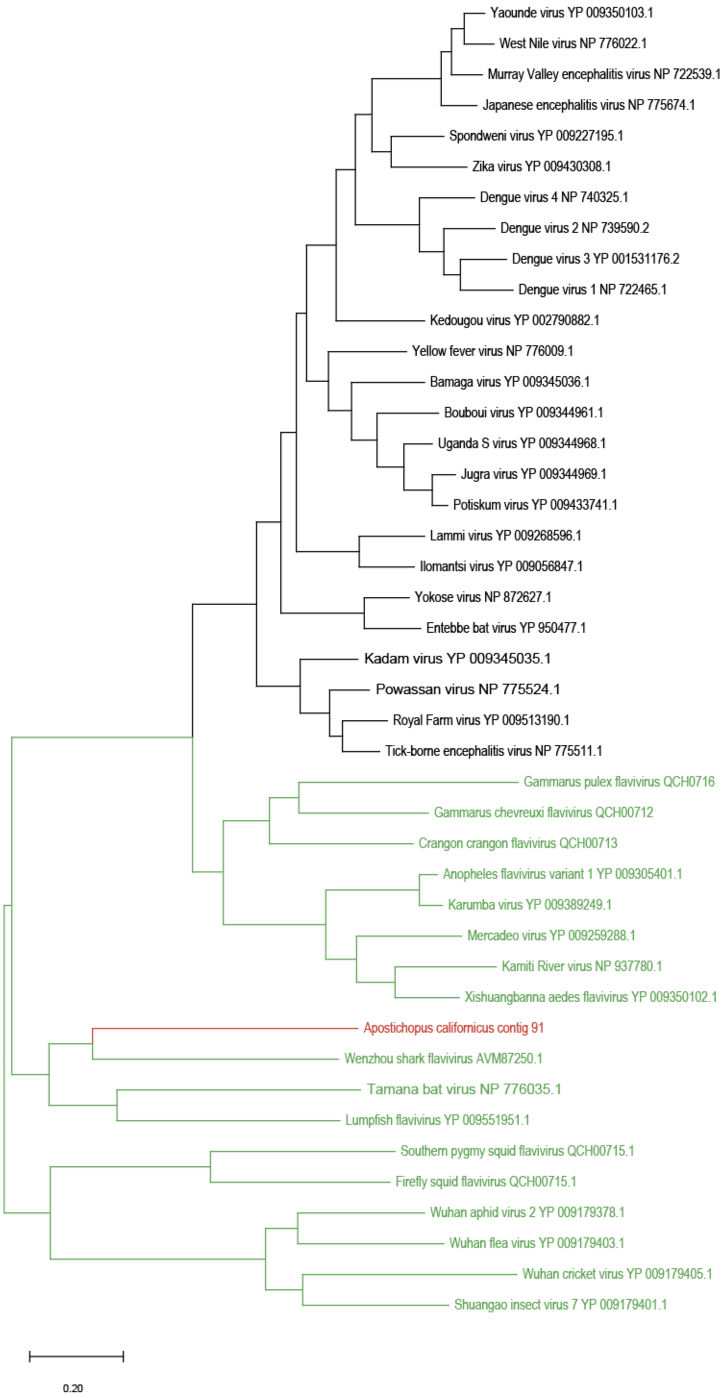

Fig. S1: Phylogenetic representation of *Apostichopus californicus* flavivirus-like contig 91. The tree was constructed by performing an alignment of overlapping regions with best BLASTx matches at NCBI using the CLC Sequence Viewer 8.0 native alignment algorithm. The tree is based on an ~420 amino acid alignment by maximum likelihood with Poisson substitution and employing the Nearest-Neighbor-Interchange heuristic method in MEGA 6.0 [1]. The green branches indicate the emerging aquatic and invertebrate-associated flavivirus clade [2].

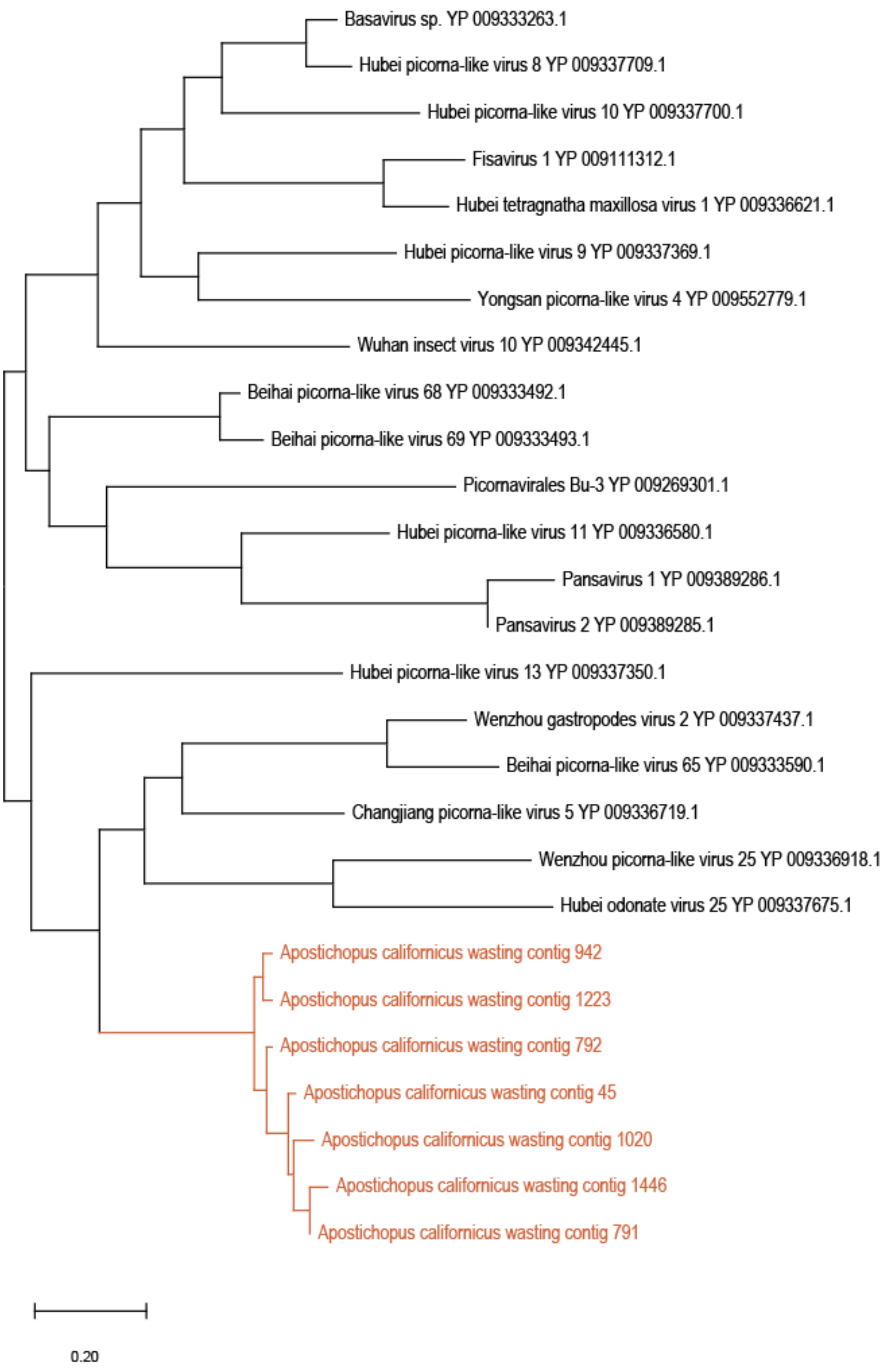

Fig. S2: Phylogenetic representation of holothurian-associated Picornavirales-like genome fragments. The tree was constructed by performing an alignment of an overlapping region (~100 amino acid) of the rhv domain with best BLASTx matches in the non-redundant database at NCBI. The tree was constructed by maximum likelihood with Poisson substitution and employing the Nearest-Neighbor-Interchange heuristic method in MEGA 6.0 [1].

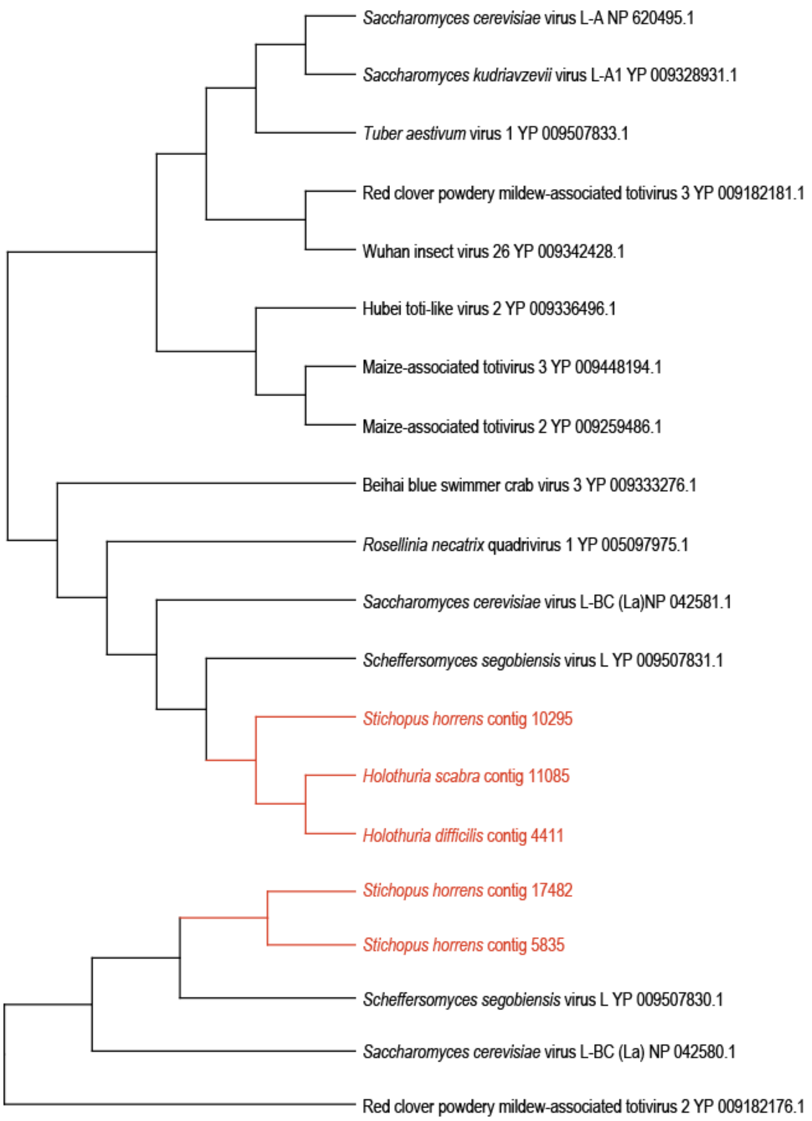

Fig. S3: Phylogenetic representations of holothurian-associated totivirus-like genome fragments. The trees were constructed by performing an alignment of an overlapping region of the RdRp (top) and Cp (bottom) domains with best BLASTx matches at NCBI. The trees are based on ~156 amino acid (for RdRp) and 87 amino acid (for Cp) alignments by maximum likelihood with Poisson substitution and employing the Nearest-Neighbor-Interchange heuristic method in MEGA 6.0 [1].

References

1. Tamura, K.; Stecher, G.; Peterson, D.; Filipski, A.; Kumar, S., MEGA6: Molecular Evolutionary Genetics Analysis version 6.0. *Mol Biol Evol* **2013,** 30, (12), 2725-9.
2. Parry, R.; Asgari, S., Discovery of Novel Crustacean and Cephalopod Flaviviruses: Insights into the Evolution and Circulation of Flaviviruses between Marine Invertebrate and Vertebrate Hosts. *Journal of Virology* **2019,** 93, (14), e00432-19.
